# Supplementary material for: From sequence to enzyme mechanism using multi-label machine learning
Source: BMC Bioinformatics. 2014 May 19;15:150. doi: 10.1186/1471-2105-15-150 (PMC4229970; doi:10.1186/1471-2105-15-150)
Supplement: Additional file 2 — Java code of ml2db. Additional file ml2db_code.tar.gz contains the Java source code to run the multi-label machine learning experiments and save the results to database. The code’s Javadoc is included. [file 1471-2105-15-150-S2.zip › additional file 2/ml2db/ml2db/doc/index-files/index-12.html]

L-Index


JavaScript is disabled on your browser.


- Overview
- Package
- Class
- Use
- Tree
- Deprecated
- Index
- Help

- Prev Letter
- Next Letter

- Frames
- No Frames

- All Classes

A B C D E F G H I J K L M N O P Q R S T U V W X 


## L

LABEL - Static variable in class main.ml2db.mulan.predict.PredictionsRecordTable


labelCombCount() - Method in class main.ml2db.mulan.mulanarff.Statistics
:   returns the HashMap containing the distinct labelsets and their
    frequencies

labelFrequency(LabelSet) - Method in class main.ml2db.mulan.mulanarff.Statistics
:   returns the frequency of a labelset in the dataset

LABELLED\_ARFF\_ID - Static variable in class main.ml2db.al.database.AlExperimentsTable
:   the table column to store the arff file id containing the labelled
    instances

LABELLED\_ATTRIBUTE\_SHAPE - Static variable in class main.ml2db.graphviz.GraphVizArffColoured


LABELLED\_ATTRIBUTES - Static variable in class main.ml2db.al.database.AlEvaluationTable


LABELLED\_CLASS\_SHAPE - Static variable in class main.ml2db.graphviz.GraphVizArffColoured


LABELLED\_CLASSES - Static variable in class main.ml2db.al.database.AlEvaluationTable


LABELLED\_INSTANCE\_SHAPE - Static variable in class main.ml2db.graphviz.GraphVizArffColoured


LABELLED\_INSTANCES - Static variable in class main.ml2db.al.database.AlEvaluationTable


LABELLED\_ROLE - Static variable in class main.ml2db.al.utility.globaldistance.GlobalPositionStore


labelNames() - Method in class main.ml2db.mulan.mulanarff.IdMultiLabelInstances


LabelRandomPicker - Class in main.ml2db.al.utility.pickers
:   Class

LabelRandomPicker() - Constructor for class main.ml2db.al.utility.pickers.LabelRandomPicker


LabelsByFreqPicker - Class in main.ml2db.al.utility.pickers.byfrequency
:   Class

LabelsByFreqPicker() - Constructor for class main.ml2db.al.utility.pickers.byfrequency.LabelsByFreqPicker


LabelsByFreqPickerTest - Class in test.ml2db.al.utility.pickers
:   Class

LabelsByFreqPickerTest() - Constructor for class test.ml2db.al.utility.pickers.LabelsByFreqPickerTest


labelSets() - Method in class main.ml2db.mulan.mulanarff.Statistics
:   returns a set with the distinct labelsets of the dataset

LinearMultiLabelNNSearch - Class in main.ml2db.al.utility.neighbours
:   Class

LinearMultiLabelNNSearch(IdMultiLabelInstances) - Constructor for class main.ml2db.al.utility.neighbours.LinearMultiLabelNNSearch


LinearNNSearchSkipInstanceItself - Class in main.ml2db.al.utility.neighbours
:   Class

LinearNNSearchSkipInstanceItself(IdMultiLabelInstances) - Constructor for class main.ml2db.al.utility.neighbours.LinearNNSearchSkipInstanceItself


LinearNNSearchSkipInstanceItselfTest - Class in test.ml2db.al.utility.neighbourhood
:   Class

LinearNNSearchSkipInstanceItselfTest() - Constructor for class test.ml2db.al.utility.neighbourhood.LinearNNSearchSkipInstanceItselfTest


listOptions() - Method in class main.ml2db.weka.JaccardDistanceStored


loadData() - Method in class main.ml2db.weka.DataSetDbLoader
:   Create a data set and load it with data from database

LoadFirstValidArff - Class in test.ml2db.dataharness
:   Class

LoadFirstValidArff() - Constructor for class test.ml2db.dataharness.LoadFirstValidArff


logEvaluation() - Method in class main.ml2db.mulan.learn.crosseval.CrossOutputManager


logEvaluation() - Method in class main.ml2db.mulan.learn.traintest.TrainTestOutputManager


logHeader() - Method in class main.ml2db.weka.DataSetWriter


logTail() - Method in class main.ml2db.weka.DataSetWriter

A B C D E F G H I J K L M N O P Q R S T U V W X

- Overview
- Package
- Class
- Use
- Tree
- Deprecated
- Index
- Help

- Prev Letter
- Next Letter

- Frames
- No Frames

- All Classes
